# Optic Discs Swelling Procrastinates Wernicke’s Encephalopathy Associated with Hyperemesis Gravidarum: A Case Report and Review of Literature

**DOI:** 10.7759/cureus.2793

**Published:** 2018-06-12

**Authors:** Lam Mun-Wei, Govindasamy Gayathri, Goh Kwang Hwee, Kanesalingam Ruban, Vasudevan Suresh Kumar, Ismail Shatriah

**Affiliations:** 1 Department of Ophthalmology, School of Medical Sciences, Universiti Sains Malaysia, Kelantan, MYS; 2 Department of Ophthalmology, Hospital Sultanah Aminah, johor bahru, MYS; 3 E1 Clinic, Regency Hospital, Johor, MYS; 4 Department of Neurology, Hospital Sultanah Aminah, johor bahru, MYS

**Keywords:** optic discs swelling, severe hyperemesis gravidarum, wernicke’s encephalopathy

## Abstract

Wernicke’s encephalopathy following severe hyperemesis gravidarum is an uncommon clinical entity. We describe a rare manifestation of optic discs swelling in a pregnant woman that has caused a diagnostic dilemma. With high index of suspicion of clinical manifestations and radiological evidences, a clinical diagnosis of Wernicke’s encephalopathy was made. Intravenous thiamine therapy was instituted, and prompt improvement of clinical signs was observed. The association of optic discs swelling and Wernicke’s encephalopathy after hyperemesis gravidarum is discussed.

## Introduction

Wernicke’s encephalopathy is a neuropsychiatric syndrome secondary to thiamine deficiency and is usually associated with chronic alcohol abuse. It can complicate hyperemesis gravidarum because of the hypermetabolic state of pregnancy [1]. It is characterized by a clinical triad of ocular signs, mental status changes and ataxia [2]. The common ocular signs are nystagmus, ophthalmoplegia and conjugate gaze palsy [3]. We report a case of hyperemesis gravidarum complicated by Wernicke’s encephalopathy with the presence of optic discs swelling that could potentially cause confusion in diagnosis.

## Case presentation

A 32-year-old primigravida was admitted at 15 weeks of gestation due to severe vomiting for two months. She had episodes of severe vomiting that required two admissions previously. She also complained of progressive blurred vision, vertigo and unsteady gait for a duration of two weeks. There was no headache, fever, pain on eye movement, hearing loss or confusion.

The patient was on a peculiar diet which consists of mainly fruits and honey. However, her condition deteriorated. She has no other medical illness before her pregnancy state.

She was clinically dehydrated and walked with an ataxic gait. There were reduced reflexes over the lower limbs. Her blood pressure was normal and she was afebrile. There was no fever or signs suggestive of meningism. All other cranial nerves examinations were intact.

**Figure 1:**
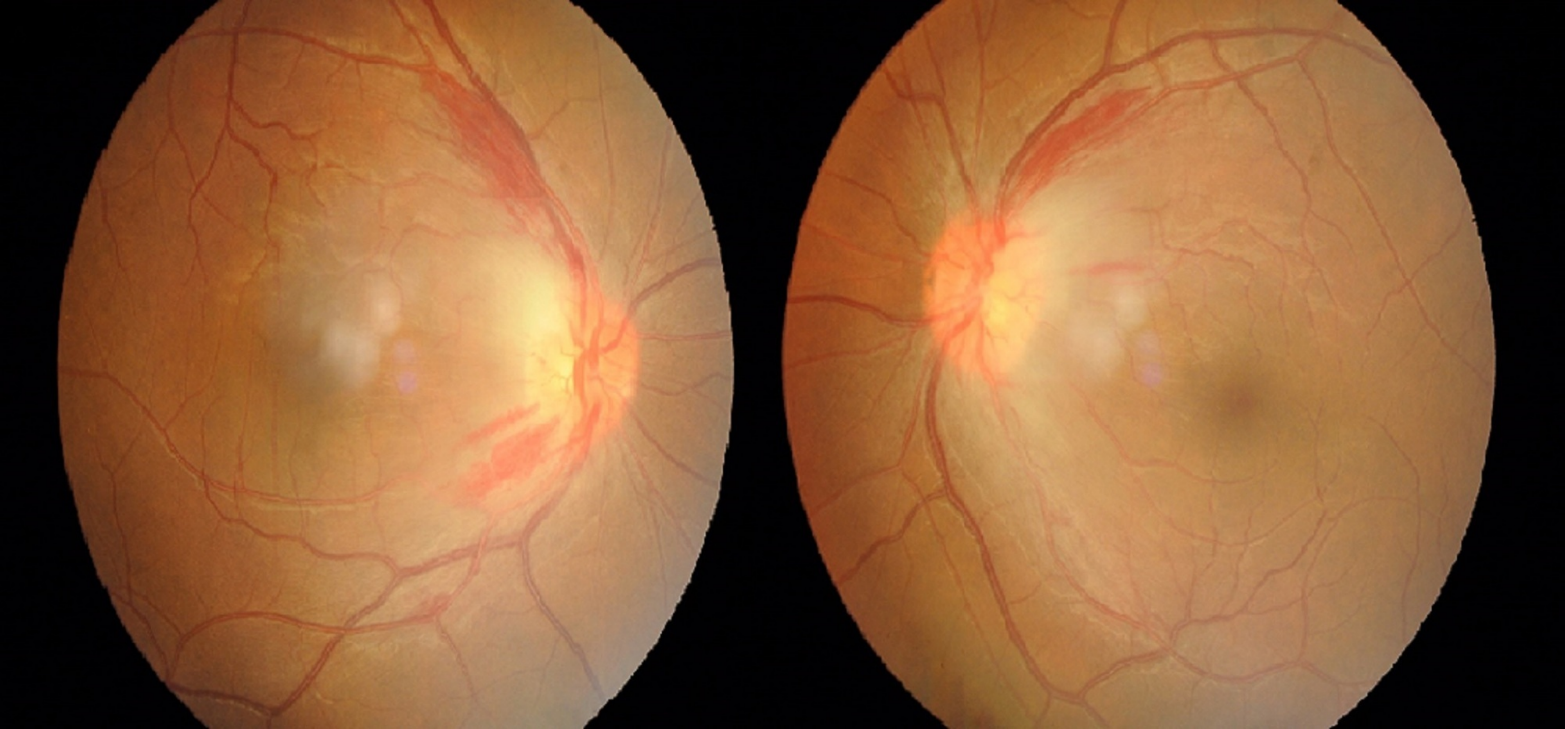
Fundus photography of bilateral eyes showing swollen and hyperemic optic discs with peripapillary hemorrhages.

Visual acuity in the right eye was 6/24 pinhole 6/18 and 6/18 pinhole 6/12 in the left eye. Bilateral horizontal nystagmus was present. There was no relative afferent pupillary defect or ophthalmoplegia observed. The anterior segment examinations of both eyes were unremarkable. The intraocular pressure was within normal range for both eyes. Fundus examination revealed bilateral swollen and hyperemic optic disc which was more marked on its temporal aspect. There were hemorrhages observed at the peripapillary retinal nerve fiber layer. There was no sign of vitritis, retinitis or choroiditis (Figure 1).

She had difficulty interpreting numbers on the pseudoisochromatic ishihara chart. There was no red desaturation or reduced light brightness in both eyes. Bedside confrontation test was grossly normal, with no obvious visual field defect detected. The optical coherence tomography of both eyes showed increased retinal nerve fiber layer (RNFL) thickness with no detectable macular edema. The RNFL thickness of the right eye was 130 μm and the left eye RNFL was 191 μm.

Blood investigations revealed low serum potassium (2.6 mmol/L), and low serum sodium (130 mmol/L). Other electrolytes, creatinine and full blood count were within normal limits. There was deranged liver function test with increased total bilirubin of 46.8 umol/L, alkaline phosphatase 74 u/L and transminitis with alanine transaminase 168 u/L. Urinary analysis showed 2+ ketonuria with no proteinuria.

**Figure 2:**
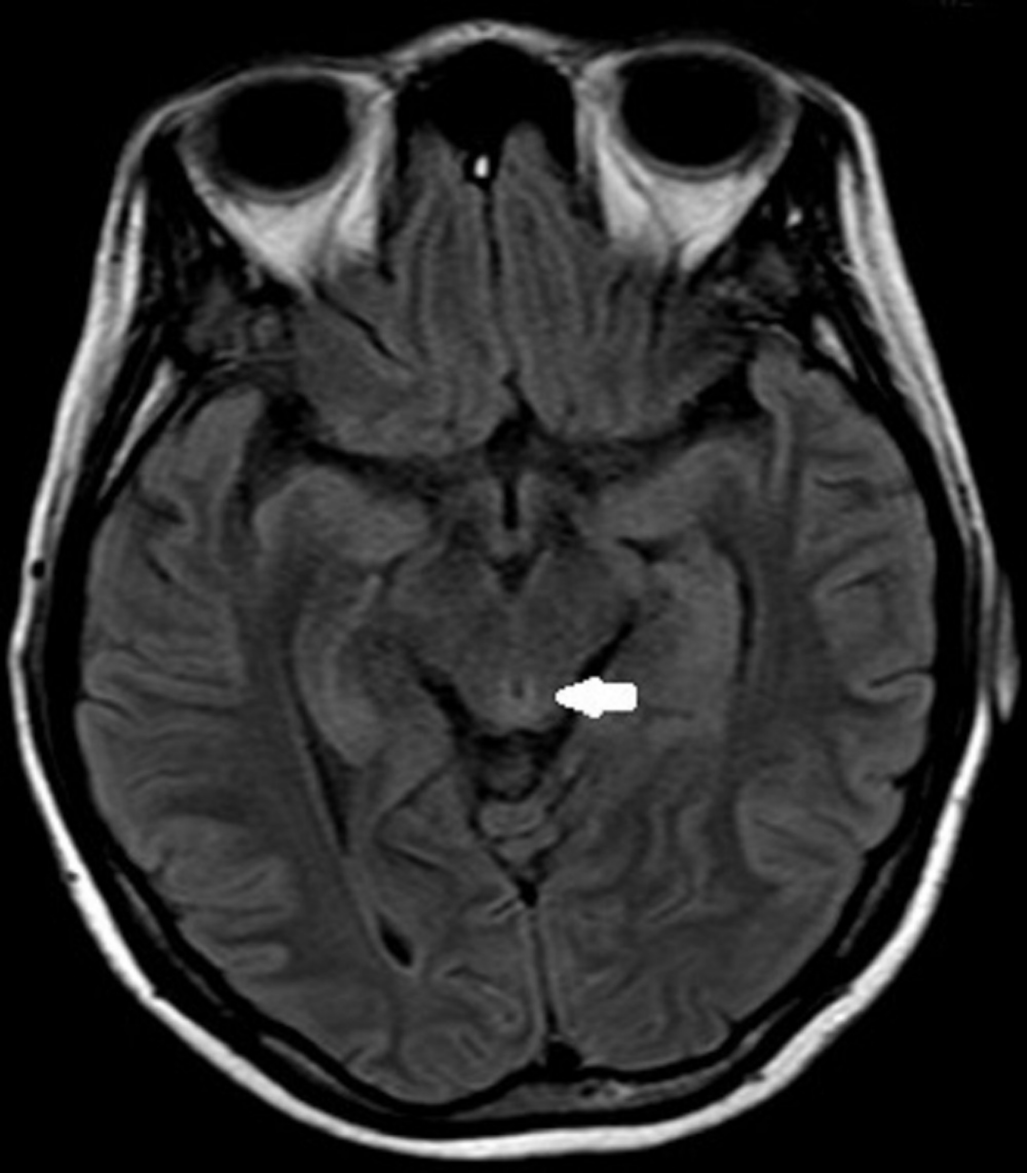
Fluid-attenuated inversion-recovery image showing mild hyperintensity in the periaqueductal area (arrows).

**Figure 3:**
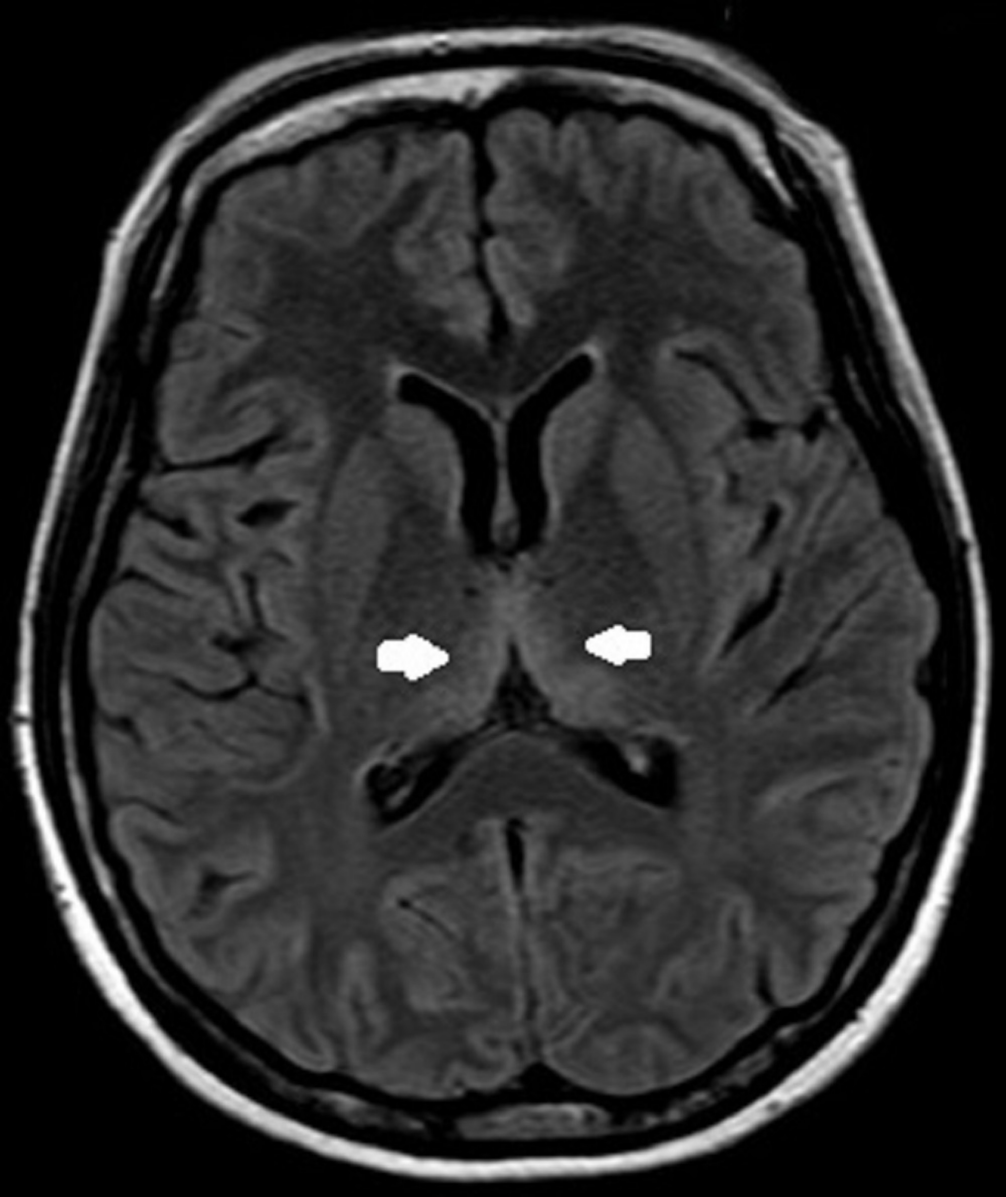
Fluid-attenuated inversion-recovery image showing symmetrical hyperintensity in the bilateral medial thalami (arrows).

**Figure 4:**
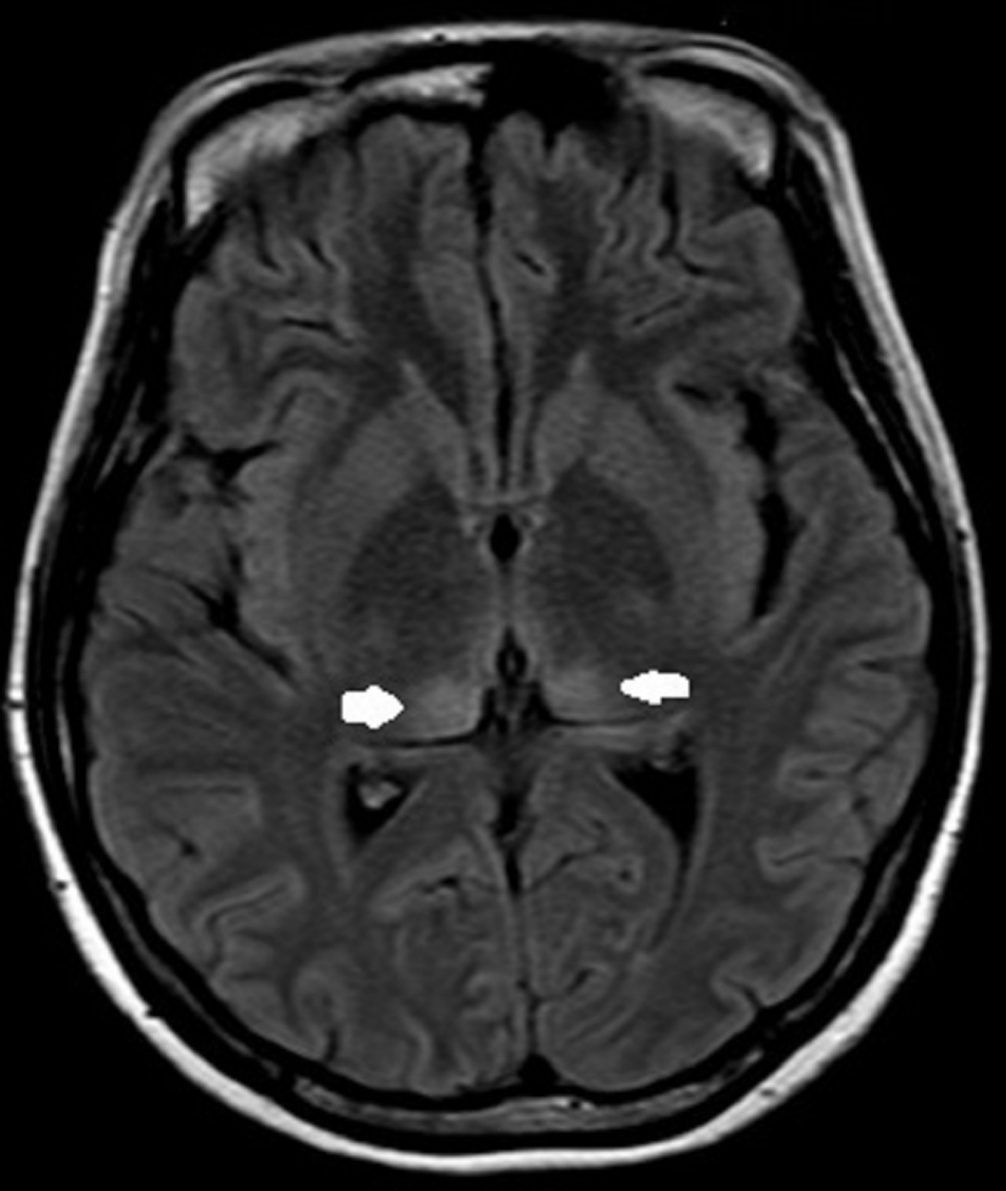
Fluid-attenuated inversion-recovery image showing symmetrical hyperintensity in the bilateral pulvinar of thalamus (arrows).

Magnetic resonance imaging of the brain showed T2 and fluid-attenuated inversion recovery (FLAIR) hyperintensity at the periaqueductal area, bilateral symmetrical medial thalamus and pulvinar of the thalamus suggestive of thiamine deficiency. There was no evidence of restricted diffusion, mass effect or midline shift. Magnetic resonance angiography and venography were within normal limits (Figures 2-4).

The patient was clinically diagnosed as Wernicke encephalopathy based on clinical and radiological evidences. She was treated empirically with high dose intravenous thiamine replacement therapy (500 mg every eight hours for three days and then 500 mg daily for five days) with intravenous fluids.

There was significant clinical improvement within the first day after initiation of thiamine therapy. She became more alert and able to respond quickly to questions. Her visual acuity improved to 6/6 for bilateral eyes. She was able to read all plates from the pseudoisochromatic ishihara chart and other optic nerve function test remained within normal limits. There was prompt resolution of nystagmus and other neurological signs. She completed the treatment for eight days as an inpatient. However, she defaulted subsequent follow-ups.

## Discussion

The triad of confusion, ocular abnormalities, and ataxia that presents in Wernicke’s encephalopathy is not obligatory in all cases, but manifested in only 47% of the patients [2]. Confusion affected 63% of the patients, ocular signs and symptoms, respectively, 96% and 57%, and ataxia occurred in 82% [2-3]. According to Chiossi et al., ocular symptoms affected 57.1% of the patients; the most frequent was diplopia and blurred vision. Ocular signs manifested in 95.9% of the study population, consisting of nystagmus, ophthalmoplegia, conjugate gaze palsy and amaurosis [2]. De Wardener and Lennox reported that only 4% had papilledema in a series of 52 Japanese prisoners-of-war who developed ocular signs in Wernicke's encephalopathy [4]. Cogan and Victor stated that retinal hemorrhages without papilledema have more frequent manifestation [5].

Bilateral eye optic disc swelling is a rare clinical manifestation in Wernicke encephalopathy. Thiamine deficiency leads to accumulation of pyruvic acid and lactic acid, and causes inadequate production of energy adenosine 5'-triphosphate. This can cause cytotoxic edema and cell death if the insult persists. There are a few explanations about the mechanism of optic discs swelling in Wernicke’s encephalopathy. An increased intracranial pressure is a known pathology. Secondly, there is a possibility of another mechanism causing an increase in pressure around the optic nerves and leading to nerve fiber swelling and obstruction of axoplasmic flow [6]. The third hypothesis is that Wernicke's encephalopathy-associated optic neuritis or neuropathy could be similar to the nutritional neuropathies [6]. Another theory of optic discs swelling with peripapillary hemorrhage in Wernicke's encephalopathy would be the classic neuropathological features of necrosis of both nerve cells and myelinated structures leading to optic nerve head edema [2].

A presumptive clinical diagnosis of Wernicke’s encephalopathy was considered in our patient based on her manifestations of nystagmus, ataxia and, in the background of severe hyperemesis gravidarum. In addition, her high sugar intake might lead to further depletion of thiamine store in the body and precipitate the onset of Wernicke’s encephalopathy. Most essentially, the patient had shown prompt improvement of ocular and neurological signs after the initiation of intravenous thiamine. Lumbar puncture was not performed in view of her dramatic improvement of clinical sign following intravenous thiamine therapy.

The magnetic resonance imaging has been reported to have sensitivity of 53% and specificity of 93% for those diagnosed with Wernicke encephalopathy [7]. Suggestive features include symmetrically increased signal intensity on FLAIR and T2 weighted images in the paraventricular areas of the thalamus, hypothalamus, mammillary bodies, periaqueductal midbrain, or cerebellum [2,7]. Our patient’s imaging showed no evidence of restricted diffusion, edema or midline shift. Magnetic resonance angiography showed normal vessels and magnetic resonance venography ruled out cerebral venous thrombosis in our patient.

Table 1 shows published cases of optic discs swelling in pregnant women with Wernicke’s encephalopathy, and our patient. Mumford, Wilson et al., Chiossi et al. and Di Gangi et al. documented deterioration in mental status when intravenous dextrose was not commenced concomitantly with intravenous thiamine [2,3,6,8]. Kantor et al. reported that their patient was not responding well to treatment probably due to central pontine myelinolysis secondary to severe electrolytes disturbances [1]. Ashraf et al. described that their patients improved with less severity but signs persisted up to one year [9]. Thus, it is mandatory to commence intravenous thiamine along with dextrose once the clinical diagnosis has been made without further delay [10]. This is very essential to prevent further depletion of thiamine store in our body by loading intravenous dextrose regime.

**Table 1:** Published case reports of optic disc swelling in pregnant woman with Wernicke’s encephalopathy. N/A: Not available

Authors	Year	Age	Gestational Week	Gravida	Ocular signs	Treatment	Outcome
Mumford [6]	1989	24	16	Gravida 2 Para 0	Nystagmus, ophthalmoplegia & papilledema	Dextrose saline followed by high dose intravenous thiamine	Significant clinical improvement in six hours. Patient became less responsive after dextrose load
Wilson et al. [8]	2006	17	19	Gravida 1 Para 0	Nystagmus, ophthalmoplegia & papilledema	Ringer lactate with dextrose followed by high dose intravenous thiamine	Clinically improved after a few days. Patient became confused after dextrose load.
Chiossi et al. [2]	2006	27	12	Gravida 4 Para 3	Nystagmus & bilateral optic discs swelling	Intravenous glucose and saline followed by intravenous thiamine	Rapid deterioration of vision and neurological signs after glucose load. Rapid improvement within one hour after thiamine given
Di Gangi et al. [3]	2012	29	20	Gravida 1 Para 0	Nystagmus & papilledema	Parenteral nutrition followed by intravenous thiamine	Deterioration of neurological signs after parenteral nutrition. Slow neurological recovery and developed Korsakoff Syndrome after lower segment caesarian section
Kantor et al. [1]	2014	25	22	Gravida 2 Para 0	Nystagmus, ophthalmoplegia & papilledema	Electively intubated and ventilated due to low Glasgow Coma Scale Vitamin, electrolytes and trace elements concomitant with intravenous thiamine	Brief clinical improvement but deteriorated with central pontine myelinolysis. Succumbed with multiorgan failure after lower segment caesarian section
Ashraf et al. [9]	2016	N/A	Second trimester	Multigravida	Nystagmus & papilledema	Intravenous thiamine and intravenous fluids	Showed less severity of clinical signs after treatment and persisting signs up to one-year duration
Our patient	2018	32	15	Gravida 1 Para 0	Nystagmus & bilateral optic discs swelling	Dextrose saline concomitant with intravenous high dose thiamine	Significant clinical improvement in one day without deterioration in mental status

## Conclusions

Persistent severe vomiting with blurring of vision in a pregnant woman is an alarming presentation to both obstetricians and ophthalmologists. Bilateral optic discs swelling with retinal hemorrhage is an uncommon finding in Wernicke encephalopathy. Early administration of intravenous thiamine is important to those pregnant women with hyperemesis gravidarum that are at risk of Wernicke encephalopathy. This condition is treatable and reversible with rapid recovery if identified early.
